# Therapeutic Maintenance of Baricitinib and Tofacitinib in Real Life

**DOI:** 10.3390/jcm9103319

**Published:** 2020-10-16

**Authors:** Valentine Deprez, Laure Le Monnier, Jean-Marc Sobhy-Danial, Franck Grados, Isabelle Henry-Desailly, Sarah Salomon-Goëb, Thibault Rabin, Sanja Ristic, Mathurin Fumery, Patrice Fardellone, Vincent Goëb

**Affiliations:** 1 Department of Rheumatology, Amiens University Hospital, 80000 Amiens, France; LeMonnier.Laure@chu-amiens.fr (L.L.M.); SobhyDanial.Jean-Marc@chu-amiens.fr (J.-M.S.-D.); Grados.Franck@chu-amiens.fr (F.G.); DesaillyHenry.Isabelle@chu-amiens.fr (I.H.-D.); Salomon.Sarah@chu-amiens.fr (S.S.-G.); Rabin.Thibault@chu-amiens.fr (T.R.); Ristic.Sanja@chu-amiens.fr (S.R.); fardellone.patrice@chu-amiens.fr (P.F.); goeb.vincent@chu-amiens.fr (V.G.); 2 Department of Gastroenterology, Amiens University Hospital, 80000 Amiens, France; fumery.mathurin@chu-amiens.fr

**Keywords:** rheumatoid arthritis, JAK inhibitors, baricitinib, tofacitinib, therapeutic maintenance

## Abstract

Background: Janus kinase inhibitors (JAKis) represent a new alternative to treat rheumatoid arthritis (RA). The objective of this study was to evaluate the effectiveness, tolerance profile, and maintenance of these treatments (tofacitinib and baricitinib) in real life. Methods: All patients in the rheumatology department of Amiens University Hospital treated by JAKis for RA were included from 1 October 2017 to 20 May 2020. Clinical and biological data were provided retrospectively in this observational and single-center study. We aimed to study the JAKi maintenance rate at 12 months and their clinical and biological safety profiles. Results: Fifty-five patients were included. Drug maintenance at 12 months was 67.6%. Factors associated with poorer maintenance were a higher Charlson comorbidity index (HR 1.311 (1.089–1.579); *p* = 0.0042), a higher age (HR 1.055 (1.015–1.096); *p* = 0.0067), and corticosteroids therapy at initiation (HR 2.722 (1.006–7.365); *p* = 0.0487). The clinical and biological safety profile was generally good. Conclusions: Our study found that a higher Charlson index, age, and corticosteroids appeared to be associated with the earlier discontinuation of treatment. JAKis had a response and tolerance profile in real life at least equivalent to that of biological disease-modifying antirheumatic drugs (bDMARDs).

## 1. Introduction

Rheumatoid arthritis (RA) is the most common chronic inflammatory rheumatic disease [1]. It can cause structural damages, reduce quality of life, and lead to permanent disability. The therapeutic management of RA is therefore essential in rheumatology research and has undergone profound changes over the past few decades. Biological agents have rapidly multiplied, and a new therapeutic class has recently emerged: Janus kinase inhibitors (JAKis).

Janus kinases (JAKs) are essential enzymes in the signaling pathways of many surface cytokine receptors, including those in the inflammatory physiology. JAKis reduce inflammation by modulating the intracellular activity of JAKs and disrupting the signaling of multiple pro-inflammatory cytokines. In France in 2017, two molecules became available in current practice: baricitinib and tofacitinib; these can be prescribed as second-line therapy after conventional synthetic disease-modifying antirheumatic drug (csDMARD) failure [2,3,4,5].

Some information about the efficacy and safety profile of these molecules in current practice are now available, but there is still a lack of data about therapeutic maintenance [6,7,8]. The European League against Rheumatism (EULAR) recommendations in 2019 also raised some concerns about the safety and effectiveness profile of using JAKis after an interleukin 6 (IL6) failure or after another JAKi failure [4].

The main objective of this study was to study JAKi persistence at 12 months as a reflection of the effectiveness and safety profile in real life. The secondary objectives were to identify the factors associated with discontinuation of JAKi therapy, the potential biological issues, the effectiveness profile, and the collection of adverse effects. 

## 2. Materials and Methods

### 2.1. Patient Selection

This observational, single-center, and retrospective study included all patients having benefited from the introduction of a JAKi for RA in the Rheumatology department of Amiens University Hospital from 1 October 2017 to 20 May 2020. The choice of treatment was left to the discretion of prescribing physicians according to the recommendations of the French Rheumatology Society [9].

All patients were informed about the study’s objectives and agreed to the anonymous use of the collected data. The study was conducted in accordance with the Declaration of Helsinki. The protocol was approved by the Ethics Committee of Department of Clinical Research and Innovation (DRCI) of the Amiens-Picardie University Hospital, and a conformity declaration to a reference methodology to the National Commission for Computing and Civil Liberties (CNIL) was made (Project identification code: PI2020_843_0024).

Inclusion criteria for the patients were:

- RA according to the 2010 ACR/EULAR criteria [10].
- Age 18 years or older.
- Initiation of JAKi therapy.
- No objection to the use of their data.

Non-inclusion criteria were a refusal to participate and patients under legal protection measures.

### 2.2. Data Recorded

Data were collected as part of routine clinical practice, and electronic medical records were obtained. For each patient, the following variables were recorded from the medical file:

- Baseline characteristics (age, sex, body mass index, history of pulmonary, digestive and urogenital infections, neoplastic history, Charlson comorbidity index [11], and smoking).
- Disease characteristics (duration of RA, positivity of rheumatoid factor (RF) and anticitrullinated protein antibodies (ACPA), presence of extra-articular manifestation, presence of erosion, prior treatments, and concomitant treatments).
- Clinical data at baseline, 3 months, 6 months, and 12 months (number of nocturnal awakenings, duration of morning rusting, pain Visual Analog Scale (VAS), patient global health VAS, disease activity score in 28 joints using erythrocyte sedimentation rate (DAS-28 ESR), disease activity score in 28 joints using C-reactive protein (DAS-28 CRP), number of swollen joints (NSJ), number of tender joints (NTJ), and secondary effects (infections, neoplasia or cardiovascular events)).
- Biological data at baseline, 3 months, 6 months, and 12 months (complete blood count (CBC), sedimentation rate (ESR), C-reactive protein (CRP), liver function, creatinine, modification of diet in renal disease (MDRD) clearance, and lipid profile including total cholesterol, triglycerides, low density lipoprotein cholesterol (LDLc), and high density lipoprotein cholesterol (HDLc)).

### 2.3. Statistical Analysis

Quantitative variables are expressed as median and quartiles, and categorical variables are expressed as number and percentage. Missing data were not replaced. Drug maintenance was analyzed by the Kaplan–Meier method. The association between treatment discontinuation and baseline characteristics was assessed using a univariate Cox model and presented in terms of a hazard ratio (HR) with 95% confidence interval (CI). *p* < 0.05 was considered statistically significant. All statistical analyses were performed using SAS software version 9.4 (SAS Institute Inc, Cary, NC, USA).

## 3. Results

### 3.1. Patient Selection and Characteristics

Among 56 patients who met the inclusion criteria, only 55 were included because one patient refused to participate. Seven patients received 5 mg of tofacitinib twice daily, and 48 patients received 4 or 2 mg of baricitinib daily. We observed that six patients (12.5%) received a daily half-dose of 2 mg due to either an age over 75 years or a moderate renal failure, according to the recommendations. Only two out of six patients were able to benefit from the maximum dose of baricitinib due to a good clinical and biological safety profile. Four patients remained on 2 mg of baricitinib daily because of persistent renal failure but with controlled rheumatism at this posology for two patients. The characteristics at baseline of the 55 patients are shown in Table 1. Due to this small sample of patients receiving tofacitinib and the similarity of their characteristics, this study analyzed both molecules together. We could notice that four patients received baricitinib and then tofacitinib with same effectiveness and safety profile (primary inefficacy of two molecules for one patient, secondary inefficacy for another patient, and digestive adverse effects for a third patient). Only one patient with the treatment failure of baricitinib was still on tofacitinib at the time of data collection, i.e., at one year and two months after its introduction. These four patients were analyzed once in the baricitinib group. 

### 3.2. JAKi Maintenance

**Figure 1:**
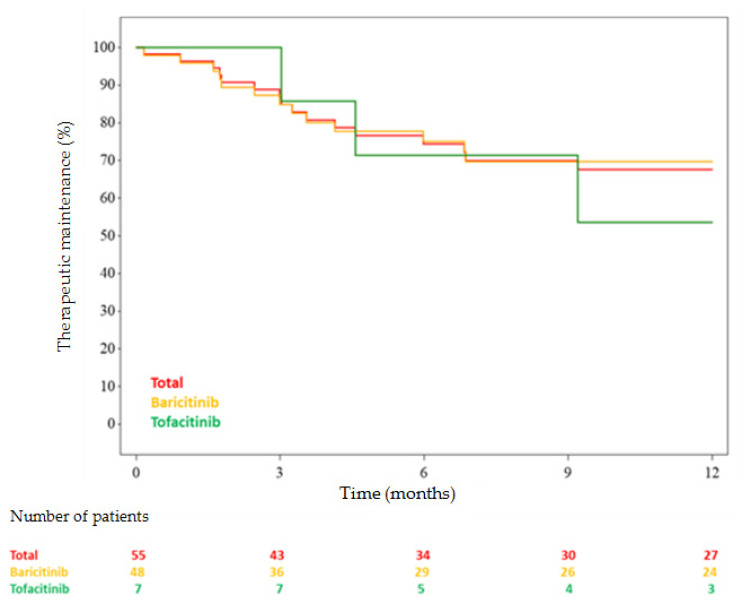
Janus kinase inhibitor maintenance in time.

The following-up median was 11.2 months (3.3–12.0). As shown in Table 2 and Figure 1, the rate of drug maintenance at 12 months was 67.6% (52.47–78.85).

### 3.3. Factors Associated with Discontinuation of JAKis and Adverse Effects

Our univariate analysis revealed three factors statistically associated with JAKi discontinuation: the Charlson comorbidity index (HR 1.358 (1.126–1.638); *p* = 0.0014), age (HR 1.055 (1.015–1.096); *p* = 0.0067), and corticosteroids at initiation (HR 2.722 (1.006–7.365); *p* = 0.0487) (Table 3). No other demographic, clinical, or paraclinical characteristics were found to be associated with drug discontinuation. 

No significant statistical link was found between therapeutic maintenance and prior treatment by IL6 inhibitors (*p* = 0.7598) either with treatment by methotrexate at initiation (*p* = 0.2330) or the absence of prior biological disease-modifying antirheumatic drugs (bDMARDs) (*p* = 0.6438). 

The reasons for JAKi discontinuation within 12 months were: primary inefficacy in seven patients (43.8%), digestive intolerance in six (37.5%), infectious adverse effects in three (18.8%), secondary inefficacy in one (6.3%), cardiovascular events in one (6.3%), and biological abnormalities in one (6.3%).

The cardiovascular events listed in our study during the 12 months follow-up were: unbalanced arterial hypertension and a myocardial infarction.

The infectious adverse effects listed in our study were: exacerbations of chronic obstructive pulmonary disease, pneumonia, recurrent upper airway infections, recurrent urinary tract infections, and recurrent herpes labialis. No herpetic zoster was recorded. 

The laboratory abnormality that led to the discontinuation of treatment was the worsening of chronic renal failure (change from stage 3 to stage 4 of chronic kidney disease).

No patient presented with neoplasia during follow-up.

### 3.4. Biological Data

The safety of JAKis was evaluated using biological data and the difference between the initiation of treatment and three and six months (Table 4). This study showed a slight decrease in hemoglobin (mean of −0.5 g/dL at six months), but we observed that the anemia present at the initiation of treatment in five patients (9.1%) was corrected for all at six months. We also found an elevation of platelets (mean of 41,032/mm^3^ at six months) and a diminution of polynuclear neutrophils (mean of −383.4/mm^3^ at six months) but no difference in liver function, renal function, or lipid profile. 

### 3.5. Effectiveness Profile

**Figure 2:**
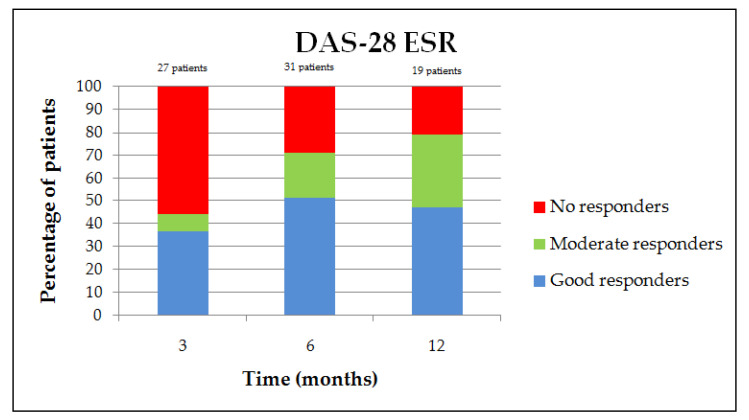
European League against Rheumatism (EULAR) response variation on DAS-28 ESR.

**Figure 3:**
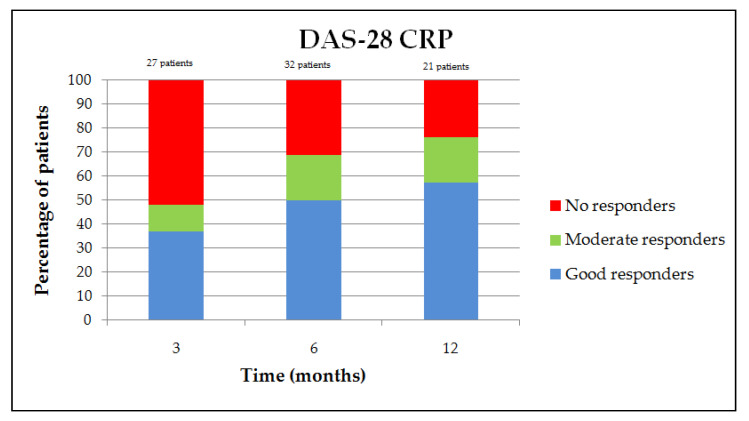
EULAR response variation on DAS-28 CRP.

**Figure 4:**
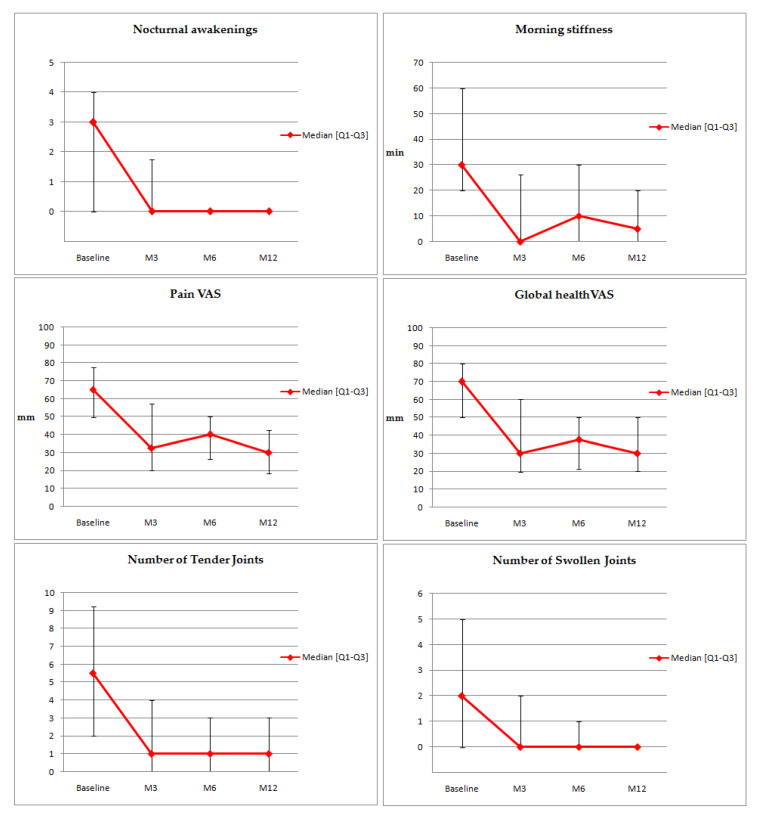
Clinical parameter variation. VAS: Visual Analog Scale.

The proportion of moderate and good responders, according to the EULAR response criteria, was on the DAS-28 ESR and CRP, respectively, 44.4% and 48.1% at 3 months, 71% and 68.8% at 6 months, and 78.9% and 76.2% at 12 months (Figure 2 and Figure 3) [12]. The clinical parameter evolution is summarized in Figure 4. There is a clinical report of improvement in a patient’s rheumatoid nodules in terms of the size and functional discomfort in the upper limbs. Regarding corticosteroid withdrawal, 23 patients (41.8% of the population) had corticosteroid therapy associated with JAKis at baseline, 20 patients were analyzed at six months, and eight of them (40%) were weaned off corticosteroids. However, the 12 patients still with corticosteroids had a decreased dosage for eight of them, with an average of 4.8 mg (median of 5 mg with a range of 1–10 mg) against a baseline mean of 8.3 mg (median of 7 mg with a range of 2–20 mg). No difference in methotrexate posology was observed in this study after JAKi initiation. 

## 4. Discussion

Our study evaluated JAKi maintenance in 55 patients with RA in routine practice in the Rheumatology department of Amiens University Hospital. We found 74.45% and 67.6% of patients continuing treatment at 6 and 12 months, respectively. The factors found to be significantly associated with drug discontinuation in univariate analysis at 12 months was a lower Charlson comorbidity index at initiation (HR 1.358 (1.126–1.638); *p* = 0.0014), age (HR 1.055 (1.015–1.096); *p* = 0.0067), and corticosteroids at initiation (HR 2.722 (1.006–7.365); *p* = 0.0487). No atypia was found in adverse events recorded, but the presence of a myocardial infarction in this small sample needs to be highlighted.

Drug maintenance at 12 months in our patients was slightly lower compared to published studies, except for a study published in May 2020 reporting a drug maintenance at 12 months of abatacept of 68% [13]. A study published in 2006 about methotrexate, a first-line treatment for RA, found a therapeutic maintenance of 76%. Another study about tocilizumab in 2017 showed a therapeutic maintenance of 82.9%, and another study about infliximab in 2008 showed a therapeutic maintenance of 78% [14,15,16]. We can also quote a study of 2019 that compared therapeutic maintenance for tocilizumab at 12 months between patients treated by intravenous tocilizumab (80%) and patients who switched for subcutaneous tocilizumab (77.7%) [17].

Our population was similar concerning baseline characteristics in comparison with patients in the RA-BEAM study about baricitinib, but our population was older with a longer duration of RA compared to the ORAL-STRATEGY study about tofacitinib [18,19]. Patients in our study had a generally lower active RA at the initiation of JAKis [18,19,20,21]. We can explain this difference by the real-life type of this study in which no severe activity of RA could be tolerated. 

We included a small number of patients treated by tofacitinib, but our study design had no influence on the choice of rheumatologist. It seems fairly intuitive to say that taking baricitinib once a day is more attractive than taking tofacitinib twice a day for patients, and baricitinib was available in France several months before tofacitinib. Additionally, a European re-evaluation of tofacitinib was carried out in May 2019 following the interim analyses of clinical study A3921133 that demonstrated an increased risk of severe venous thromboembolism compared to tumor necrosis factor inhibitors at a dosage of 10 mg twice daily [22]. This posology is indicated in the gastroenterological setting for treatment of ulcerative colitis but not of RA. However, there may be a greater concern about the use of this molecule compared to baricitinib that may explain this difference in our study. 

Regarding the biological follow-up between initiation and three and six months, we did not find any major difference in the hemoglobin level (median differences of −0.4 and −0.8 g/dL, respectively). However, we noticed that the anemia observed at baseline in 9.1% of patients was corrected at six months for all. Conversely, anemia appeared in 9.4% of patients at six months. Though the initial anemia etiology was not investigated, we can speculate that its disappearance was due to the correction of the biological inflammatory syndrome but also by the direct action of the JAKis, which can influence the process of hematopoiesis [23,24,25]. Erythropoietin, as well as growth factors of the white line, acts to obtain physiological effects through the JAK system and, in particular, JAK2. The contradiction between correction of anemia and creation of anemia in this study is therefore explained by the fact that we studied two molecules with slightly different modes of action, baricitinib being a more significant inhibitor of JAK2 than tofacitinib. However, finding a correction of anemia at six months for all patients in our population has a clinical interest because anemia is one of the factors of chronic asthenia expressed by a majority of patients regardless of disease control [26,27].

Regarding the adverse events listed in this study, we identified a myocardial infarction and an antral ulcer. Regarding the myocardial infarction, this was a patient with other cardiovascular risk factors, and all studies have been reassuring about the cardiovascular risk of JAKis. This isolated case does not challenge the available large-scale data [7,8]. Likewise, there was a case of antral ulcers in a patient with concomitant use of non-steroidal anti-inflammatory drugs. This type of adverse event is known and described in the literature under JAKis [28]. The main hypothesis regarding the physiology of digestive perforations is that the inhibition of the JAK–STAT system is partly responsible for the inhibition of IL6 signaling, and digestive perforations are a known and frequent risk with IL6 inhibitors. 

Our study found a higher Charlson comorbidity index as a statistically significant factor associated with lower therapeutic maintenance (HR 1.358 (1.126–1.638); *p* = 0.0014). Age, that is part of the Charlson index, and treatment by corticosteroids at baseline were also factors that were statistically associated with lower therapeutic maintenance in our univariate analysis. We could therefore suggest that, according to our study, a higher patient’s Charlson index at the start of treatment is associated with a greater probability of stopping treatment. In other words, the introduction of JAKis in patients with numerous co-morbidities should be discussed. This original sighting has never been described, to our knowledge, in JAKi treatments. Likewise, another Japanese study from 2014 showed a poorer response to biological DMARDs with a higher Charlson index in terms of DAS-28 evolution and quality of life [29]. Conversely, a German study from 2014 found no association between the Charlson index and therapeutic maintenance with adalimumab, etanercept, and infliximab [30]. Our study did not find any statistical difference of therapeutic maintenance for patients with a history of IL6 inhibitor therapy, for patients treated by JAKis with methotrexate, or for patients without prior bDMARDs.

Our study had several limitations. The retrospective characteristic induced a large variation in the duration of treatment, and many patients were censured. The small size of our single-center cohort did not allow us to perform multivariate analysis. Therefore, the association between characteristics at baseline and therapy discontinuation must be interpreted with caution and should be confirmed in multicenter studies of larger scales and longer follow-ups.

## 5. Conclusions

In view of our study, JAKis had a response and tolerance profile in real life at least equivalent to that of biological disease-modifying antirheumatic drugs. To optimize their prescription, it is suggested to take the patient’s age, his Charlson comorbidity index, and any received corticosteroids therapy into account.

Our study did not find any difference in therapeutic maintenance whether JAKis were administered first-line after the failure of methotrexate, after a history of IL6 inhibitor treatment, or in combination with methotrexate, but these data must be confirmed by other teams on a larger scale.

## Figures and Tables

**Table 1:** Baseline characteristics of the study population.

	Whole Population (*n* = 55)
Female sex, *n* (%)	45 (81.8%)
Age (years), *med [Q1–Q3]*	58.0 [46.0–67.0]
Body mass index (kg/m²), *med [Q1–Q3]*	25.9 [22.9–30.5] *
Disease duration (years), *med [Q1–Q3]*	11.0 [4.0–18.0]
Personal medical history	
Infection, *n* (%)	18/49 (36.7%)
Neoplasia, *n* (%)	4 (7.3%)
Smoking, *n* (%)	12/49 (24.5%)
Charlson comorbidity index, *med [Q1*–*Q3]*	2.0 [0.0–3.0]
DAS-28 ESR, *med [Q1*–*Q3]*	4.2 [3.5–4.9]
DAS-28 CRP, *med [Q1*–*Q3]*	4.2 [3.3–4.8]
Extra-articular manifestations, *n* (%)	8/53 (15.1%)
Treatment	
Methotrexate at initiation, *n* (%)	30 (54.5%)
Methotrexate dose (mg/week), *med (min*–*max)*	20.0 (7.5–25.0)
Corticosteroids at initiation	23 (41.8%)
Corticosteroids dose (mg/day), *med (min*–*max)*	7 (2–20)
RF positive, *n* (%)	44 (80.0%)
ACPA positive, *n* (%)	43 (78.2%)
Erosion presence, *n* (%)	34 (61.8%)

*N*: number; *med*: median value; *Q1*: first quartile; *Q3*: third quartile; RF: rheumatoid factor; ACPA: anticitrullinated protein antibodies; DAS-28 ESR: disease activity score in 28 joints using erythrocyte sedimentation rate; DAS-28 CRP: disease activity score in 28 joints using C-reactive protein; * missing data for 21 patients.

**Table 2:** Therapeutic maintenance of Janus kinase inhibitors.

	Total	Baricitinib	Tofacitinib
Stop/Censure	16/39	13/35	3/4
	Kaplan–Meier (CI 95%)		
3 months	86.89% [74.43–93.53]	84.9% [70.88–92.51]	100.0% [100.0–100.0]
6 months	74.45% [59.99–84.33]	75.1% [59.41–85.43]	71.43% [25.82–91.98]
9 months	69.93% [55.0–80.74]	69.74% [53.4–81.29]	71.43% [25.82–91.98]
12 months	67.6% [52.47–78.85]	69.74% [53.4–81.29]	53.57% [13.2–82.5]

**Table 3:** Factors associated with therapy discontinuation.

Parameters	HR	Lower 95% CI	Upper 95% CI	*p*-Value
Age (for 1 year)	1.055	1.015	1.096	0.0067
Female vs. male sex	1.851	0.420	8.164	0.4159
Disease duration (for 1 year)	1.021	0.964	1.083	0.4763
Personal medical history				
Infections	1.264	0.438	3.645	0.6647
Neoplasia	0.806	0.106	6.108	0.8349
Smoking	0.390	0.089	1.717	0.2132
Charlson comorbidity index (for 1 point)	1.358	1.126	1.638	0.0014
DAS28-ESR at initiation (for 1 point)	1.324	0.882	1.987	0.1753
DAS28-CRP (at initiation (for 1 point)	1.091	0.709	1.680	0.6925
Prior treatment				
No prior biological treatment	0.705	0.160	3.102	0.6438
1 tumor necrosis factor inhibitor versus none	0.573	0.143	2.296	0.4316
2 or more tumor necrosis factor inhibitors versus none	1.151	0.346	3.830	0.8190
Interleukin 6 inhibitor	1.167	0.434	3.137	0.7598
Rituximab	1.498	0.520	4.315	0.4541
Abatacept	0.955	0.358	2.551	0.9273
Corticosteroids at initiation	2.722	1.006	7.365	
Methotrexate at initiation	0.547	0.203	1.474	0.2330
RF and/or ACPA positive	1.318	0.299	5.809	0.7151
Erosion presence	1.116	0.405	3.072	0.8317

RF: rheumatoid factor; ACPA: anticitrullinated protein antibodies; DAS-28 ESR: disease activity score in 28 joints using erythrocyte sedimentation rate; DAS-28 CRP: disease activity score in 28 joints using C-reactive protein.

**Table 4:** Biological evolution between baseline, 3 months, and 6 months.

	Difference between Baseline and 3 Months	Difference between Baseline and 6 Months
Hemoglobin (g/dL), *med [Q1–Q3]*	−0.4 [−1.0–0.1] **	−0.8 [−1.1–0.1] *
Platelets (/mm^3^), *med [Q1*–*Q3]*	56,000 [6000–82,000] *	38,500 [−12,000–76,000]
Leukocytes (/mm^3^), *med [Q1*–*Q3]*	−300.0 [−1800–500] *	−105.0 [−1410–420]
Polynuclear neutrophils (/mm^3^), *med [Q1*–*Q3]*	−488.5 [−1229.5–516.5] *	85.5 [−1040–655]
Eosinophilic cells (/mm^3^), *med [Q1*–*Q3]*	−40.5 [−91–145] *	−24.0 [−91–−1.0]
Lymphocytes (/mm^3^), *med [Q1*–*Q3]*	41.0 [−400–792] *	−181.5 [–501–430]
ESR, *med [Q1*–*Q3]*	0.0 [−4.0–4.0] *	−0.5 [−5.0–3.0]
CRP, *med [Q1*–*Q3]*	−1.1 [−5.9–0.0] *	−3.1 [−7.2–0.0]
Aspartate transaminase (UI/L), *med [Q1*–*Q3]*	3.0 [−3.0–7.0] **	4.0 [−2.0–6.0]
Alanine transaminase (UI/L), *med [Q1*–*Q3]*	1.0 [−31.0–24.0] **	0.0 [−6.0–7.0]
Creatinine (µmol/L), *med [Q1*–*Q3]*	2.6 [−2.5–7.0] **	0.0 [−4.5–7.9]
Creatinine clearance (mL/min), *med [Q1*–*Q3]*	−2.3 [−7.8–7.3] **	0.0 [−11.0–7.7]
Total cholesterol (g/L), *med [Q1*–*Q3]*	0.1 [−0.2–0.2] ^†^	0.1 [−0.1–0.2] ^†^
Triglycerides (g/L), *med [Q1*–*Q3]*	0.0 [−0.6–0.2] ^†^	−0.3 [−0.6–0.2] ^†^
LDL cholesterol (g/L), *med [Q1*–*Q3]*	0.0 [−0.2–0.2] ^†^	0.0 [−0.1–0.1] ^†^
HDL cholesterol (g/L), *med [Q1*–*Q3]*	0.0 [−0.4–0.4] ^†^	0.0 [0.0–0.1] ^†^

*med*: median value; *Q1*: first quartile; *Q3*: third quartile; ESR: erythrocyte sedimentation rate; CRP: C-reactive protein; LDL: low-density lipoprotein; HDL: high-density lipoprotein; * missing data for 7 patients; ** missing data for 10 patients; ^†^ missing data for 25 patients.
